# Hospital quality reporting and improvement in quality of care for patients with acute myocardial infarction

**DOI:** 10.1186/s12913-018-3330-4

**Published:** 2018-07-04

**Authors:** Hayato Yamana, Mariko Kodan, Sachiko Ono, Kojiro Morita, Hiroki Matsui, Kiyohide Fushimi, Tomoaki Imamura, Hideo Yasunaga

**Affiliations:** 1 Department of Health Services Research, Graduate School of Medicine, The University of Tokyo, 7-3-1 Hongo, Bunkyo-ku, Tokyo, 113-0033 Japan; 2 Department of Clinical Data Management and Research, Clinical Research Center, National Hospital Organization Headquarters, 2-5-21 Higashigaoka, Meguro-ku, Tokyo, 152-8621 Japan; 3 Department of Health Policy and Informatics, Tokyo Medical and Dental University Graduate School of Medicine, 1-5-45 Yushima, Bunkyo-ku, Tokyo, 113-8510 Japan; 4 Department of Biostatistics & Bioinformatics, The University of Tokyo, 7-3-1 Hongo, Bunkyo-ku, Tokyo, 113-0033 Japan; 5 Department of Clinical Epidemiology and Health Economics, School of Public Health, The University of Tokyo, 7-3-1 Hongo, Bunkyo-ku, Tokyo, 113-0033 Japan; 6 Department of Public Health, Health Management and Policy, Nara Medical University, 840 Shijo-cho, Kashihara, Nara, 634-0813 Japan

**Keywords:** Quality improvement, Mortality, Cardiovascular diseases

## Abstract

**Background:**

Although public reporting of hospital performance is becoming common, it remains uncertain whether public reporting leads to improvement in clinical outcomes. This study was conducted to evaluate whether enrollment in a quality reporting project is associated with improvement in quality of care for patients with acute myocardial infarction.

**Methods:**

We conducted a quasi-experimental study using hospital census survey and national inpatient database in Japan. Hospitals enrolled in a ministry-led quality reporting project were matched with non-reporting control hospitals by one-to-one propensity score matching using hospital characteristics. Using the inpatient data of acute myocardial infarction patients hospitalized in the matched hospitals during 2011–2013, difference-in-differences analyses were conducted to evaluate the changes in unadjusted and risk-adjusted in-hospital mortality rates over time that are attributable to intervention.

**Results:**

Matching between hospitals created a cohort of 30,220 patients with characteristics similar between the 135 reporting and 135 non-reporting hospitals. Overall in-hospital mortality rates were 13.2% in both the reporting and non-reporting hospitals. There was no significant association between hospital enrollment in the quality reporting project and change over time in unadjusted mortality (OR, 0.98; 95% CI, 0.80–1.22). In 28,168 patients eligible for evaluation of risk-adjusted mortality, enrollment was also not associated with change in risk-adjusted mortality (OR, 0.98; 95% CI, 0.81–1.17).

**Conclusions:**

Enrollment in the quality reporting project was not associated with short-term improvement in quality of care for patients with acute myocardial infarction. Additional efforts may be necessary to improve quality of care.

**Electronic supplementary material:**

The online version of this article (10.1186/s12913-018-3330-4) contains supplementary material, which is available to authorized users.

## Background

Public reporting of hospital performance data is becoming increasingly common worldwide [1]. Release of performance data is designed to increase transparency and accountability [2], and theoretically leads to improvement in quality of care through two pathways: selection of better-performing providers and change in patterns of care [3]. However, studies have also suggested unintended and negative consequences of public reporting, such as avoidance of treating severe patients [4–6]. Therefore, evaluation of the effects of public reporting is important for deciding what, how, and whether to report.

Observational studies that assessed the association between reporting of hospital performance data and improvement in clinical outcomes have produced inconsistent results [4, 7–10]. In a cluster randomized trial, public release of quality indicators did not significantly improve quality of care for patients with acute myocardial infarction (AMI) or congestive heart failure [11]. Overall, there is insufficient evidence to conclude that public reporting leads to improvement in clinical outcomes [12, 13].

Recent large-scale quasi-experimental studies from the United States have added to the literature by reporting no significant association between enrollment in a quality improvement program and improvement in clinical outcomes of Medicare beneficiaries or patients hospitalized in academic hospitals [14, 15]. Meanwhile, evidence is lacking for other programs and patient populations. In Japan, a quality reporting project led by the Ministry of Health, Labour and Welfare (MHLW) involving hospital groups was introduced in 2010 [16, 17]. The objectives were to improve quality of care and to promote quality reporting in Japan. Nationwide hospital group organizations participating in the project were to select quality indicators (QIs), collect and summarize data from multiple participating hospitals within each group, and publicly report the QIs using their websites or other means. The MHLW required that process and outcome QIs for specific diseases (e.g., cancer, stroke, AMI, and diabetes), patient safety, and regional cooperation were selected, and that organizations reported their results and suggestions for improvements to the MHLW. Each year, 2 to 3 organizations were selected for partial funding by the MHLW, after which they could continue the reporting at their own expense. However, the effectiveness of the project remains unclear.

In the present study, we evaluated the association between enrollment in the Japanese quality reporting project and improvement in quality of care for patients with AMI. We hypothesized that enrollment in the project would lead to improvements in outcome indicators (unadjusted mortality, risk-adjusted mortality, and mortality of patients undergoing percutaneous coronary intervention [PCI]) and a process indicator (treatment with aspirin within 2 days of admission). Propensity score matching by national hospital census data was used to select control hospitals for comparison, and difference-in-differences analyses using data from a nationwide administrative database were conducted.

## Methods

The study was approved by the Institutional Review Board of The University of Tokyo (approval number: 3501). Because of the anonymous nature of the data, the need for informed consent was waived.

### Data source

In Japan, a lump-sum payment system was introduced in acute-care hospitals from 2003. The Diagnosis Procedure Combination (DPC) database is a national administrative database for patients admitted to hospitals with implementation of the payment system (DPC hospitals) [18]. Participation in the database is mandatory for academic hospitals and voluntary for community hospitals. Participating hospitals provide administrative claims and abstract discharge data for all their acute-care inpatients. The database includes the following information: hospital identification code; patient demographic and clinical information; admission and discharge statuses; main and secondary diagnoses; surgeries and procedures performed; medications; and special reimbursements for specific conditions. Diagnoses are recorded using International Classification of Diseases, Tenth Revision (ICD-10) codes. Suspected diagnoses are allowed to be recorded, and are designated as such. Surgeries, drugs, procedures, and special reimbursements are coded according to the Japanese fee schedule for reimbursement, and their daily use or application is recorded. Clinical information recorded in the database includes the Killip class, a classification for AMI patients based on physical signs of heart failure (class I to class IV in ascending order of severity) [19, 20].

The Reporting System for Functions of Medical Institutions is a census survey of hospitals in Japan initiated in 2014 [21]. It includes detailed structural information for institutions such as location, hospital type (DPC hospitals divided into category 1 [university hospitals], category 2 [community hospitals with equivalent functions to university hospitals], and category 3 [other DPC hospitals], and non-DPC hospitals), numbers of acute-care and long-term-care beds, numbers of nurses and physical therapists, and number of imaging devices. Process information including numbers of inpatients, ambulance acceptances, and out-of-hours hospitalizations is also recorded. In addition, electronically recorded claims data are used to identify monthly numbers of specific procedures performed in each institution, including operations, mechanical ventilation, and renal replacement therapy.

### Hospital and patient selection

Using the DPC database, patients hospitalized for AMI (confirmed main diagnosis with ICD-10 codes I21.x) between July 2010 and March 2014 were searched, and hospitals with at least 10 AMI hospitalizations between July 2010 and March 2011 were identified. The websites of organizations participating in the reporting project were also searched to identify hospitals that reported performance data in fiscal years 2011, 2012, and 2013. Fiscal years in Japan start in April and end in March. Enrollment statuses for the 3 years and data from the 2014 Reporting System for Functions of Medical Institutions were linked with the DPC data. DPC category 1 hospitals (university hospitals) were excluded because university hospitals were rare among the hospitals affiliated to the organizations participating in the reporting project. Hospitals that were no longer categorized as DPC hospitals according to the 2014 data and hospitals that discontinued reporting during the study period were also excluded. Reporting hospitals were defined as those that started reporting in any year from 2011 through 2013 and non-reporting hospitals were defined as those without reporting in all 3 years. The restrictions of the DPC database prohibited direct contact with the participating hospitals. Thus, a survey on the details of each hospital’s reporting program and implementation status was not conducted.

### Variables

The hospital-level characteristics examined in the study are presented in Table 1. In Japan, there is a local area division based on medical services supply termed the Secondary Medical Area [21]. The annual number of ambulance acceptances within the Secondary Medical Area associated with each hospital were summarized and used as a regional characteristic. In addition to the characteristics obtained from the hospital survey data, the July 2010–March 2011 DPC data were used to identify the number of AMI patients and whether there were hospitalizations to intensive care units (ICUs) and emergency centers, and whether hospitals conducted coronary artery bypass graft (CABG) surgery. As a variable for performance prior to introduction of the quality reporting project, each hospital’s risk-adjusted mortality rate for AMI patients, as proposed by the Quality Indicator/Improvement Project (QIP) [22], were calculated. QIP risk-adjusted mortality takes into account age, sex, and Killip class for risk adjustment. In the derivation of risk-adjusted mortality rate, patients from all hospitals with at least 10 cases of AMI identified in the DPC database in the 6-month period were included to calculate the overall mortality.

**Table 1:** Hospital characteristics before and after propensity score matching

Hospital characteristic	All hospitals			Matched hospitals		
	Reporting, n (%) (*N* = 146)	Non-reporting, n (%) (*N* = 327)	Standardized difference	Reporting, n (%) (*N* = 135)	Non-reporting, n (%) (*N* = 135)	Standardized difference
Geographical region						
Hokkaido and Tohoku	12 (8.2)	55 (16.8)	−26.2	12 (8.9)	15 (11.1)	−7.4
Kanto	31 (21.2)	87 (26.6)	−12.6	31 (23.0)	28 (20.7)	5.4
Chubu	26 (17.8)	55 (16.8)	2.6	24 (17.8)	23 (17.0)	2.0
Kansai	37 (25.3)	50 (15.3)	25.2	31 (23.0)	31 (23.0)	0.0
Chugoku and Shikoku	14 (9.6)	22 (6.7)	10.5	12 (8.9)	11 (8.1)	2.7
Kyushu	26 (17.8)	58 (17.7)	0.2	25 (18.5)	27 (20.0)	−3.8
DPC category 2 hospital	25 (17.1)	42 (12.8)	12.0	20 (14.8)	21 (15.6)	−2.1
Number of acute-care beds						
< 300	31 (21.2)	89 (27.2)	−14.0	31 (23.0)	36 (26.7)	−8.6
300–399	37 (25.3)	92 (28.1)	−6.3	34 (25.2)	33 (24.4)	1.7
400–499	27 (18.5)	58 (17.7)	2.0	27 (20.0)	24 (17.8)	5.7
≥ 500	51 (34.9)	88 (26.9)	17.4	43 (31.9)	42 (31.1)	1.6
Annual number of hospital ambulance acceptances						
< 2000	30 (20.5)	96 (29.4)	−20.5	30 (22.2)	33 (24.4)	−5.3
2000–2999	34 (23.3)	82 (25.1)	−4.2	32 (23.7)	34 (25.2)	−3.4
3000–3999	25 (17.1)	50 (15.3)	5.0	22 (16.3)	22 (16.3)	0.0
≥ 4000	57 (39.0)	99 (30.3)	18.5	51 (37.8)	46 (34.1)	7.7
Three or more angiography systems	37 (25.3)	74 (22.6)	6.4	35 (25.9)	28 (20.7)	12.3
Annual number of regional ambulance acceptances						
< 10,000	20 (13.7)	86 (26.3)	−31.9	19 (14.1)	20 (14.8)	−2.1
10,000–29,999	57 (39.0)	105 (32.1)	14.5	54 (40.0)	55 (40.7)	−1.5
30,000–49,999	30 (20.5)	47 (14.4)	16.3	26 (19.3)	24 (17.8)	3.8
≥ 50,000	39 (26.7)	89 (27.2)	−1.1	36 (26.7)	36 (26.7)	0.0
ICU admission of AMI patients	73 (50.0)	121 (37.0)	26.4	65 (48.1)	64 (47.4)	1.5
Emergency center admission of AMI patients	31 (21.2)	44 (13.5)	20.6	21 (15.6)	28 (20.7)	−13.5
CABG surgery for AMI patients	58 (39.7)	96 (29.4)	21.9	49 (36.3)	44 (32.6)	7.8
Hospital volume of AMI patients						
< 20	29 (19.9)	75 (22.9)	−7.5	29 (21.5)	31 (23.0)	−3.6
20–34	34 (23.3)	83 (25.4)	−4.9	32 (23.7)	28 (20.7)	7.1
35–49	32 (21.9)	73 (22.3)	−1.0	29 (21.5)	30 (22.2)	− 1.8
≥ 50	51 (34.9)	96 (29.4)	12.0	45 (33.3)	46 (34.1)	−1.6
Risk-adjusted mortality	0.127 ± 0.068	0.127 ± 0.073	0.1	0.124 ± 0.066	0.129 ± 0.078	−7.8

*Abbreviations:* *AMI* Acute myocardial infarction, *CABG* Coronary artery bypass graft, *DPC* Diagnosis Procedure Combination, *ICU* intensive care unit

The main outcomes of the study were 2 types of mortality in AMI patients: unadjusted in-hospital mortality and QIP risk-adjusted in-hospital mortality. The in-hospital mortality of patients admitted by ambulance and subsequently treated with PCI in particular were also evaluated. As an indicator of process, treatment of AMI patients with aspirin within 2 days of admission was examined. The indicators were selected from the QIs determined by the National Hospital Organization and the QIP. The inclusion/exclusion criteria and risk adjustment methods for the indicators are presented in Additional file 1: Table S1.

### Statistical analysis

To adjust for differences in hospital characteristics between the reporting and non-reporting hospitals, one-to-one propensity score matching of hospitals [23] was performed. To estimate the propensity score, a logistic regression model with enrollment in the reporting project as a dependent variable was first fitted. Hospital and regional characteristics and risk-adjusted mortality in the 6 months prior to 2011, presented in Table 1, were entered as independent variables. The c-statistic was used to evaluate the discriminatory ability of the logistic regression model. Using the estimated propensity scores, nearest neighbor matching without replacement, within 0.2 times the standard deviation of the estimated propensity scores, was conducted. The standardized difference was used to compare the characteristics between the two groups before and after matching [24, 25]. An absolute standardized difference of > 10 was considered indicative of imbalance.

The data for AMI patients hospitalized in the matched hospitals from April 2011 to March 2014 were then used to conduct difference-in-differences analyses. Non-reporting hospitals were operationally assigned the same enrollment year as their matched reporting counterparts, and patients hospitalized before the initiation of reporting were excluded. Logistic regression models predicting the outcomes were fitted, with the following entered as independent variables: reporting status of the admitted hospital (reporting vs. non-reporting); year when the reporting was started (as a continuous variable); a yearly time variable representing the number of years after hospital enrollment (difference between year of patient hospitalization and hospital enrollment year, as a continuous variable); and an interaction term multiplying the reporting status and the post-enrollment time variable. Analyses were performed with adjustment for clustering within hospitals using cluster-robust standard errors. The c-statistic was used to evaluate the discriminatory ability of the logistic regression model. The difference-in-differences approach is an econometric method used to evaluate the effect of a policy change by isolating the change in outcome over time related to the intervention from the change experienced over time without the intervention [26, 27]. In this study, the interaction term of the reporting status and the post-enrollment time variable represented the influence per year exerted by the reporting on the outcomes after enrollment, considering the year when reporting was started as the baseline. Enrollment year was entered in the models to adjust for pre-enrollment trends.

In addition to the main analysis, 3 further analyses were conducted with the same regression model used for the main analysis and selection of patients hospitalized in different time periods. First, to examine whether there were changes in outcomes immediately following enrollment, a difference-in-differences analysis between the year when reporting was started and the year before that was performed (2-year, before-after analysis). Second, patients hospitalized in the year before enrollment were added to the main analysis, assuming that quality improvement occurs immediately in the year of enrollment and that the amount of improvement stays the same thereafter. In these analyses, hospitals that started reporting in 2011 and their matched counterparts were excluded because of the lack of full-year data in 2010. Third, the main analysis was repeated excluding hospitals that started reporting in 2013, the final year of observation.

A 2-sided *P* value of < 0.05 was considered significant. Statistical analyses were conducted using Stata/MP version 14.0 (StataCorp, College Station, TX, USA).

## Results

### Hospital characteristics and propensity score matching

Two hundred hospitals reported hospital performance in 2011 under the MHLW project. Subsequently, the participating hospitals increased to 327 in 2012 and 438 in 2013. Both DPC and non-DPC hospitals participated in the project.

From the DPC database, 28,548 AMI patients admitted between July 2010 and March 2011 were identified. Among these patients, 27,597 were from 613 hospitals with at least 10 admissions.

**Fig. 1:**
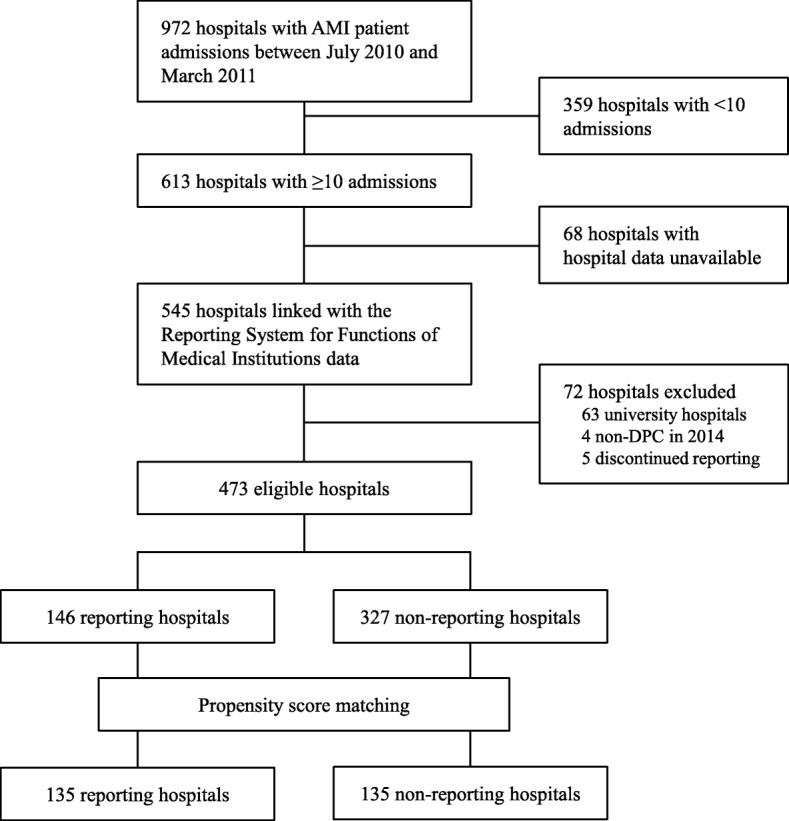
Hospital selection. AMI, acute myocardial infarction; DPC, Diagnosis Procedure Combination

The flow of hospital selection is presented in Fig. 1. After linkage with the Reporting System for Functions of Medical Institutions data and exclusion of hospitals, 146 hospitals that participated in the reporting project (48, 69, and 29 hospitals started reporting in 2011, 2012, and 2013, respectively) and 327 non-reporting hospitals were identified. There were 21,004 AMI admissions from these 473 hospitals in the 6-month period. The hospital characteristics before matching are presented in Table 1. The reporting hospitals were larger and better-equipped than the non-reporting hospitals, and admitted more AMI patients.

Propensity score matching between the reporting and non-reporting hospitals produced 135 pairs of hospitals. The c-statistic was 0.667. These hospitals admitted 12,516 AMI patients in the 6-month period. The hospital characteristics of the matched hospitals are also presented in Table 1. Most of the characteristics were balanced between the 2 groups of hospitals. Of the 135 matched reporting hospitals, 42, 66, and 27 hospitals started reporting in 2011, 2012, and 2013, respectively.

### Patient characteristics and outcomes after hospital matching

Overall, 30,220 AMI hospitalizations during the April 2011–March 2014 period from the 270 matched hospitals were included in the analysis of mortality, and 3998 of these patients (13.2%) died during hospitalization. The inclusion criteria for QIP risk-adjusted mortality reduced the number of eligible patients to 28,168 for risk-adjusted mortality analysis, and there were 3330 deaths (11.8%). Table 2 presents the characteristics of the patients admitted to the reporting and non-reporting hospitals during different time periods. At 2 years after the start of reporting, the reporting hospitals admitted fewer patients categorized as Killip class 1 compared with the non-reporting hospitals. Otherwise, patients admitted to the reporting hospitals had similar patient characteristics to those admitted to the matched non-reporting hospitals. There were 13,370 AMI patients admitted by ambulance and treated with PCI, and 276 deaths (2.1%) among these patients. Aspirin was administered within 2 days of admission in 22,655 of 26,989 patients who met the inclusion criteria for the process QI (83.9%). The outcomes among the patients admitted to the reporting and non-reporting hospitals during different time periods are presented in Table 3. There were no apparent difference in outcomes between the reporting and non-reporting hospitals.

**Table 2:** Characteristics of patients admitted to the matched reporting and non-reporting hospitals

Patient characteristic	Year 0			Year 1			Year 2			All		
	Reporting, n (%) (*N* = 6919)	Non-reporting, n (%) (*N* = 6170)	SD	Reporting, n (%) (*N* = 5267)	Non-reporting, n (%) (*N* = 5115)	SD	Reporting, n (%) (*N* = 2345)	Non-reporting, n (%) (*N* = 2352)	SD	Reporting, n (%) (*N* = 14,531)	Non-reporting, n (%) (*N* = 13,637)	SD
Sex												
Male	5044 (72.9)	4440 (72.0)	2.1	3922 (74.5)	3709 (72.5)	4.4	1681 (71.7)	1685 (71.6)	0.1	10,647 (73.3)	9834 (72.1)	2.6
Female	1875 (27.1)	1730 (28.0)	−2.1	1345 (25.5)	1406 (27.5)	−4.4	664 (28.3)	667 (28.4)	−0.1	3884 (26.7)	3803 (27.9)	− 2.6
Age, years												
18–64	2456 (35.5)	2097 (34.0)	3.2	1871 (35.5)	1710 (33.4)	4.4	768 (32.8)	819 (34.8)	−4.4	5095 (35.1)	4626 (33.9)	2.4
65–74	1822 (26.3)	1615 (26.2)	0.4	1389 (26.4)	1390 (27.2)	−1.8	626 (26.7)	637 (27.1)	−0.9	3837 (26.4)	3642 (26.7)	−0.7
75–84	1760 (25.4)	1633 (26.5)	− 2.3	1362 (25.9)	1323 (25.9)	0.0	652 (27.8)	579 (24.6)	7.3	3774 (26.0)	3535 (25.9)	0.1
≥ 85	881 (12.7)	825 (13.4)	−1.9	645 (12.2)	692 (13.5)	−3.8	299 (12.8)	317 (13.5)	−2.2	1825 (12.6)	1834 (13.4)	−2.6
Killip class												
Class 1	2878 (41.6)	2683 (43.5)	−3.8	2120 (40.3)	2300 (45.0)	−9.5	895 (38.2)	1082 (46.0)	−15.9	5893 (40.6)	6065 (44.5)	−7.9
Class 2	1937 (28.0)	1727 (28.0)	0.0	1531 (29.1)	1347(26.3)	6.1	730 (31.1)	649 (27.6)	7.8	4198 (28.9)	3723 (27.3)	3.5
Class 3	671 (9.7)	550 (8.9)	2.7	438 (8.3)	439 (8.6)	−1.0	198 (8.4)	185 (7.9)	2.1	1307 (9.0)	1174 (8.6)	1.4
Class 4	878 (12.7)	816 (13.2)	−1.6	700 (13.3)	661 (12.9)	1.1	298 (12.7)	300 (12.8)	−0.1	1876 (12.9)	1777 (13.0)	− 0.4
Missing	555 (8.0)	394 (6.4)	6.3	478 (9.1)	368 (7.2)	6.9	224 (9.6)	136 (5.8)	14.2	1257 (8.7)	898 (6.6)	7.8

*Abbreviation: SD* standardized difference

**Table 3:** Outcomes of patients admitted to the matched reporting and non-reporting hospitals

Year	Unadjusted mortality, %		Risk-adjusted mortality, %		Mortality of ambulance-admitted PCI patients, %		Aspirin within 2 days of admission, %	
	Reporting	Non-reporting	Reporting	Non-reporting	Reporting	Non-reporting	Reporting	Non-reporting
0	13.4	13.2	12.2	12.1	2.1	1.9	82.5	83.4
1	12.8	13.2	10.9	11.8	1.9	2.1	85.8	84.7
2	13.6	13.5	12.1	11.9	3.0	1.7	83.3	84.4
All	13.2	13.2	11.7	11.9	2.2	2.0	83.8	84.1

*Abbreviation:* *PCI* percutaneous coronary intervention

### Difference-in-differences analyses

The results of the logistic regression analyses are presented in Table 4. For all 4 indicators, the odds ratios for reporting status were not significant, indicating that hospital performances were similar between the reporting and non-reporting hospitals in the baseline years. In addition, the baseline performances did not differ significantly across the years that reporting was started. For all 4 indicators, the interaction term was not significant, indicating that reporting was not associated with changes in quality of care. The results of the 3 sensitivity analyses are presented in Table 5. In all 3 analyses, the interaction terms were not significant. The c-statistics of the models in the main analysis and the 3 additional analyses are presented in Additional file 1: Table S2.

**Table 4:** Results of the difference-in-differences logistic regression analyses

Variable	Unadjusted mortality		Risk-adjusted mortality		Mortality of PCI patients admitted by ambulance		Aspirin within 2 days	
	OR (95% CI)	*P*	OR (95% CI)	*P*	OR (95% CI)	*P*	OR (95% CI)	*P*
Reporting hospital	1.01 (0.83–1.24)	0.916	0.99 (0.78–1.24)	0.905	0.98 (0.65–1.49)	0.933	0.97 (0.73–1.23)	0.815
Year that reporting started	1.01 (0.87–1.18)	0.870	1.07 (0.89–1.28)	0.467	1.03 (0.77–1.37)	0.861	1.05 (0.86–1.27)	0.637
Years after start of reporting	1.02 (0.89–1.17)	0.819	1.01 (0.90–1.13)	0.868	0.99 (0.74–1.32)	0.920	1.07 (0.93–1.23)	0.327
Interaction term^a^	0.98 (0.80–1.22)	0.879	0.98 (0.81–1.17)	0.789	1.18 (0.83–1.66)	0.351	1.03 (0.81–1.30)	0.826

^a^The interaction term of the reporting status and the post-enrollment time variable represents the influence per year exerted by reporting on the outcomes

*Abbreviations*: *CI* confidence interval, *OR* odds ratio, *PCI* percutaneous coronary intervention

**Table 5:** Results of the additional difference-in-differences logistic regression analyses

Analysis	Unadjusted mortality		Risk-adjusted mortality		Mortality of PCI patients admitted by ambulance		Aspirin within 2 days	
	OR (95% CI)	*P*	OR (95% CI)	*P*	OR (95% CI)	*P*	OR (95% CI)	*P*
2-year, before-after	0.90 (0.72–1.12)	0.354	0.83 (0.63–1.08)	0.160	0.89 (0.46–1.72)	0.734	0.87 (0.64–1.20)	0.402
Addition of year before enrollment	0.90 (0.78–1.05)	0.185	0.85 (0.71–1.01)	0.060	0.81 (0.57–1.16)	0.246	0.97 (0.80–1.16)	0.706
Exclusion of hospitals enrolled in 2013	1.00 (0.81–1.23)	0.981	0.94 (0.79–1.12)	0.506	1.19 (0.84–1.68)	0.322	1.05 (0.83–1.32)	0.706

The interaction term of the reporting status and the post-enrollment time variable are presented

*Abbreviations*: *CI* confidence interval, *OR* odds ratio, *PCI* percutaneous coronary intervention

## Discussion

The present study utilized 2 large databases to evaluate the association between enrollment in the hospital quality reporting project in Japan and improvement in quality of care for patients with AMI. Within the study period, enrollment was associated with neither improvement in process of care nor improvement in outcomes. Reporting of QIs alone may not be sufficient to achieve short-term improvement in quality of care.

There are 2 key assumptions for difference-in-differences analyses: parallel trends and common shocks [26, 27]. Selection of an appropriate control group to meet these assumptions is challenging, and matching is recommended when treatment and control groups differ in pre-intervention levels or trends [27]. In the present study, participation of hospitals in the reporting project was not randomized, and imbalance between the reporting and non-reporting hospitals was an expected result. For example, large hospitals may allocate more resources to improve their quality of care. In addition, an institution’s role in the regional healthcare system may influence whether or not it participates in the project. We obtained detailed hospital characteristics from the census survey data for hospitals and conducted propensity score matching using hospital and regional variables. After the propensity score matching, the characteristics were well-balanced between the reporting and non-reporting hospitals. Furthermore, the numbers of patients and their characteristics were similar across the patients hospitalized in the matched hospitals. Although it cannot be tested whether the 2 assumptions hold true, these results suggest that the matching created valid comparison groups.

Using the data for patients admitted to the 270 matched hospitals, we examined 4 measures of quality of care. Reporting was not associated with change in mortality of AMI patients, and this finding was observed for both unadjusted and risk-adjusted mortality. Limiting the patients to those who underwent PCI after arrival by ambulance did not change this finding. We also examined a process indicator for AMI patients. Although not significant, there was a small increase over years in the probability of receiving aspirin within 2 days of admission. However, there was no apparent increase that could be attributed to the reporting status of hospitals. Some previous observational studies reported improvements in quality of care after the introduction of quality reporting [8, 10]. However, these studies either did not account for secular trends or their control groups had insufficient comparability. More rigorously designed studies showed no association between enrollment in a quality reporting program in the United States and improvement in quality of surgical care [14, 15]. The present study showed similar results for AMI patients in Japan.

Multiple investigators suggest the potential to avoid treating severe patients as one of the unintended and negative consequences of public reporting [4–6]. To evaluate whether this phenomenon existed, we compared the numbers and backgrounds of the treated patients between the matched reporting and non-reporting hospitals. Over years after participation in the project, there was no apparent difference in the numbers of treated AMI patients between the matched reporting and non-reporting hospitals. Furthermore, the patient backgrounds remained generally similar between the 2 groups over years. As an exception, the proportion of patients with less severe AMI decreased in the reporting hospitals. These results suggest that unintended consequences of public reporting, such as avoidance of treating more severe patients, were unlikely.

Previous studies on hospital quality reporting were primarily conducted in the United States, and the present study adds to the body of evidence by reporting results from hospitals operating under a different situation. The healthcare system in Japan is characterized by universal insurance coverage and a nationally uniform fee schedule for reimbursement [28, 29]. In addition, primary care physicians play little role as gatekeepers and patients have free access to virtually all hospitals [29–31]. Competition may make hospitals sensitive to their performance and motivate them to improve their quality of care. However, in the present study, there was no evidence of a short-term incremental effect of the reporting. QI reporting alone may be insufficient, and additional efforts may be necessary to improve quality of care. Further research is required to achieve *kaizen* in healthcare.

Several limitations of the present study should be considered. First, we used administrative data for both hospital matching and outcome assessments. There could be unmeasured hospital characteristics that act as confounders, and the matching may not be perfect. Also, measures of quality depended on outcomes, processes, and risk factors that were obtainable from the DPC database. Second, precise information for each hospital’s quality reporting program was unobtainable, and it was unclear whether the program was actually carried out. Likewise, the control hospitals may have implemented original quality improvement programs, either as a spillover effect or as an independent attempt. Third, the observation period after the start of the program was 3 years at most. The long-term effect of the reporting program was unobservable in this study. Lastly, AMI was the focus of this study. Further research is required to determine the effect of quality reporting on other conditions.

## Conclusions

Enrollment in the ministry-led quality reporting project in Japan was not associated with short-term improvement in quality of care of patients with AMI. Further research is required to identify additional efforts that could improve quality of care.

## Additional file

Additional file 1: **Table S1.** Inclusion/exclusion criteria and risk adjustment methods for the quality indicators used as outcomes. **Table S2.** C-statistics of logistic regression models predicting the outcomes. (DOC 42 kb)

## Abbreviations

AMI: acute myocardial infarction
CABG: coronary artery bypass graft
DPC: Diagnosis Procedure Combination
ICD-10: International Classification of Diseases, Tenth Revision
ICU: intensive care unit
MHLW: Ministry of Health, Labour and Welfare
PCI: percutaneous coronary intervention
QI: quality indicator
QIP: Quality Indicator/Improvement Project
