# Supplementary material for: Hospital quality reporting and improvement in quality of care for patients with acute myocardial infarction
Source: BMC Health Serv Res. 2018 Jul 4;18:523. doi: 10.1186/s12913-018-3330-4 (PMC6033287; doi:10.1186/s12913-018-3330-4)
Supplement: Supplementary file 1 — Table S1. Inclusion/exclusion criteria and risk adjustment methods for the quality indicators used as outcomes. Table S2. C-statistics of logistic regression models predicting the outcomes. (DOC 42 kb) [file 12913_2018_3330_MOESM1_ESM.doc]

**Additional file 1**

**Table S1** Inclusion/exclusion criteria and risk adjustment methods for the quality indicators used as outcomes

| Quality indicator | Inclusion criteria | Exclusion criteria | Risk adjustment |
| --- | --- | --- | --- |
| Unadjusted mortality | Main diagnosis:  I21.x | None | None |
| Risk-adjusted mortality  (QIP #4003) | Age ≥18 years  Main diagnosis:  I21.x, I22.x  Emergency admission | Unknown discharge site  Cardiac arrest on admission  (I46.9) | Sex  Age  (18–64, 65–74, 75–85, >85)  Killip class  (1, 2, 3, 4, unknown) |
| Mortality of PCI patients admitted by ambulance  (NHO #15) | Main diagnosis:  I21.x, I22.x, I24.x, I20.0  Admission by ambulance  Admission from home  PCI within 2 days | Cardiac arrest on admission  (I46.9)  Killip class 3 or 4 | None |
| Aspirin within 2 days  (QIP #0474) | Main diagnosis:  I21.x | Length of stay ≤2 days | None |

Diagnoses are expressed using International Classification of Diseases, Tenth Revision codes.

Abbreviations: NHO, National Hospital Organization; PCI, percutaneous coronary intervention; QIP, Quality Indicator/Improvement Project.

**Table S2** C-statistics of logistic regression models predicting the outcomes

| Analysis | Outcomes | | | |
| --- | --- | --- | --- | --- |
|  | Unadjusted mortality | Risk-adjusted mortality | Mortality of PCI patients admitted by ambulance | Aspirin within 2 days |
| Main analysis | 0.502 | 0.887 | 0.523 | 0.515 |
| Additional analysis 1: 2-year, before-after | 0.512 | 0.889 | 0.544 | 0.519 |
| Additional analysis 2: addition of year before enrollment | 0.513 | 0.890 | 0.543 | 0.516 |
| Additional analysis 3: exclusion of hospitals enrolled in 2013 | 0.505 | 0.887 | 0.521 | 0.512 |

Abbreviations: PCI, percutaneous coronary intervention.
